# Draft Genome Sequence of Klebsiella pneumoniae UMB7779, Isolated from the Female Urinary Tract

**DOI:** 10.1128/MRA.00396-20

**Published:** 2020-05-14

**Authors:** Pavan Bhimalli, Taylor Miller-Ensminger, Adelina Voukadinova, Alan J. Wolfe, Catherine Putonti

**Affiliations:** a Department of Biology, Loyola University Chicago, Chicago, Illinois, USA; b Bioinformatics Program, Loyola University Chicago, Chicago, Illinois, USA; c Department of Microbiology and Immunology, Stritch School of Medicine, Loyola University Chicago, Maywood, Illinois, USA; d Department of Computer Science, Loyola University Chicago, Chicago, Illinois, USA; University of Southern California

## Abstract

Klebsiella pneumoniae is a pathogenic bacterium commonly responsible for urinary tract infections (UTIs). Here, we report the draft genome sequence of K. pneumoniae strain UMB7779, isolated from catheterized urine of a woman with a recurrent UTI.

## ANNOUNCEMENT

Klebsiella pneumoniae is an opportunistic pathogen that can colonize mucosal surfaces and can be community or hospital acquired (1). It can cause pneumonia, septicemias, soft tissue infections, and urinary tract infections (UTIs) (1–3). In fact, the urinary tract is the most common site of infection for this species, and *K. pneumonia* accounts for 2 to 6% of hospital-acquired UTIs and 4.3 to 7% of community-acquired UTIs (4). This species is known to be problematic in a clinical setting, as it is resistant to a wide array of antibiotics and capable of evading host immune responses (5, 6). We recently isolated K. pneumoniae UMB7779 from a catheterized urine sample from a female patient with a recurrent UTI.

K. pneumoniae UMB7779 was isolated from a previously approved institutional review board (IRB) study (University of California, San Diego, IRB no. 170077AW) using the expanded quantitative urinary culture (EQUC) protocol (7). The sample was collected from a patient at the Women’s Pelvic Medicine Center at the University of California, San Diego, in August 2017. We used matrix-assisted laser desorption ionization–time of flight (MALDI-TOF) mass spectrometry to determine the genus and species of our isolate, following a protocol previously described (7), prior to storage at −80°C. Using aseptic techniques, a sterile inoculation loop of the sample was streaked onto a Columbia nalidixic acid (CNA) agar plate using the quadrant streak method. The plate was incubated for 24 h at 35°C with 5% CO_2_. A single colony was picked and grown overnight in LB liquid medium, and DNA was extracted using the Qiagen DNeasy blood and tissue kit. Modifications were made from the kit’s Gram-positive protocol. Two hundred thirty microliters of lysis buffer (180 μl of 20 mM Tris-Cl, 2 mM sodium EDTA, and 1.2% Triton X-100 and 50 μl of lysozyme) was added to the pellet. Step 5 of the protocol was also amended; the sample was incubated at 56°C for only 10 min. DNA was quantified using the Qubit fluorometer. The extracted DNA was sent to the Microbial Genomic Sequencing Center (MiGS) at the University of Pittsburgh for sequencing. The DNA was fragmented using an Illumina tagmentation enzyme, and indices were attached via PCR. The library was sequenced using an Illumina NextSeq 550 flow cell. This produced 2,217,322 pairs of 150-bp reads. Our raw reads were then trimmed using Sickle v1.33 (https://github.com/najoshi/sickle) and assembled using SPAdes v3.13.0 with the “only-assembler” option for k values of 55, 77, 99, and 127 (8). The genome coverage was calculated using BBMap v38.47 (https://sourceforge.net/projects/bbmap/). PATRIC v3.6.3 and the NCBI Prokaryotic Genome Annotation Pipeline (PGAP) v4.11 were used to annotate the genome sequences (9, 10). Default settings were used for all software unless noted otherwise.

The K. pneumoniae UMB7779 draft genome is 5,571,379 bp assembled in 105 contigs. The GC content is 57.2%, with a genome coverage of 97× and an *N*_50_ score of 136,300 bp. PGAP predicted 5,383 protein-coding regions, 5 rRNA operons, and 79 tRNAs. PATRIC annotations identified genes associated with resistance to tetracycline, with several antibiotic susceptibilities predicted. Future analysis of K. pneumoniae will further expand our knowledge of this pathogen, allowing us to better understand the female urobiome and future treatment options.

### Data availability.

This whole-genome shotgun (WGS) project has been deposited in GenBank under accession no. JAAUVZ000000000. The version described in this paper is the first version, JAAUVZ010000000. The raw sequencing reads have been deposited in the SRA under accession no. SRR11441030.
